# Expression of the SNARE Protein SNAP-23 Is Essential for Cell Survival

**DOI:** 10.1371/journal.pone.0118311

**Published:** 2015-02-23

**Authors:** Sunil Kaul, Sharad K. Mittal, Lionel Feigenbaum, Michael J. Kruhlak, Paul A. Roche

**Affiliations:** 1 Experimental Immunology Branch, National Cancer Institute, National Institutes of Health, Bethesda, Maryland, United States of America; 2 Leidos Biomedical Research, Frederick National Laboratory for Cancer Research, Frederick, Maryland, United States of America; UPR 3212 CNRS -Université de Strasbourg, FRANCE

## Abstract

Members of the SNARE-family of proteins are known to be key regulators of the membrane-membrane fusion events required for intracellular membrane traffic. The ubiquitously expressed SNARE protein SNAP-23 regulates a wide variety of exocytosis events and is essential for mouse development. Germline deletion of SNAP-23 results in early embryonic lethality in mice, and for this reason we now describe mice and cell lines in which SNAP-23 can be conditionally-deleted using Cre-lox technology. Deletion of SNAP-23 in CD19-Cre expressing mice prevents B lymphocyte development and deletion of SNAP-23 using a variety of T lymphocyte-specific Cre mice prevents T lymphocyte development. Acute depletion of SNAP-23 in mouse fibroblasts leads to rapid apoptotic cell death. These data highlight the importance of SNAP-23 for cell survival and describe a mouse in which specific cell types can be eliminated by expression of tissue-specific Cre-recombinase.

## Introduction

Proteins and membranes move from compartment to compartment in eukaryotic cells by a complex process of vesicle-mediated transport and fusion. There are many discrete proteins that regulate the specificity of vesicle docking and fusion with distinct target membranes, and some of these proteins are members of the SNARE family of integral membrane proteins [1]. According to the “classic” definition, there are SNARE proteins on vesicles themselves (v-SNAREs) and on the target membranes for these vesicles (termed t-SNAREs). Formation of a ternary complex of v-SNAREs with t-SNAREs leads to membrane fusion, thereby delivering cargo molecules from a donor vesicle to a target membrane for a wide variety of intracellular transport processes such as intra-Golgi transport, endosome-to-lysosome transport, and regulated exocytosis from intracellular storage vesicles to the plasma membrane.

The t-SNARE family consists of a family of homologous Syntaxin proteins (each of which reside on distinct intracellular target membranes) that bind to a “common” t-SNARE protein of the SNAP-25 family. SNAP-25 is neuron/neuroendocrine cell-specific, and germline deletion of *Snap25* results in viable mouse embryos that appear “normal”, however these mice die immediately after birth because synaptic vesicle exocytosis (and action potential propagation) is completely absent [2]. There is another SNAP-25 family member, termed SNAP-23, which is also expressed in the brain, but SNAP-23 is not expressed at pre-synaptic nerve terminals [3] and thus the presence of SNAP-23 in *Snap25*-null mice does not rescue the synaptic vesicle exocytosis defect. SNAP-23 regulates a wide-variety of regulated- and constitutive-exocytosis events in diverse cell types including adipocytes [4], mast cells [5,6], platelets [7,8], pancreatic acinar cells [9], kidney epithelial cells [10], and neutrophils [11].

We have previously generated mice harboring a germline deletion of *Snap23* and found that mouse embryos lacking an intact SNAP-23 gene were not viable past e3.5 [12]. Although this finding is consistent with the hypothesis that SNAP-23 expression is essential for cell viability, our inability to obtain viable embryos lacking SNAP-23 left open the possibility that SNAP-23 was essential for a specific step in embryonic development and was not more generally required for cell survival. For this reason we have now generated BAC transgenic mice on a SNAP-23-null background in order to allow us to conditionally delete SNAP-23 in distinct cell types to assess the importance of SNAP-23 in cell differentiation/survival in the context of a living mouse. We now report that deletion of SNAP-23 by expressing Cre in distinct cell types results in the death of the Cre expressing cell. Furthermore, acute deletion of SNAP-23 in SNAP-23-floxed mouse embryonic fibroblasts (MEFs) results in rapid apoptotic death of the Cre-expressing MEF, revealing an essential role for the SNARE protein SNAP-23 in cell survival.

## Results and Discussion

### Generation of a SNAP-23-floxed mouse

**Fig 1:**
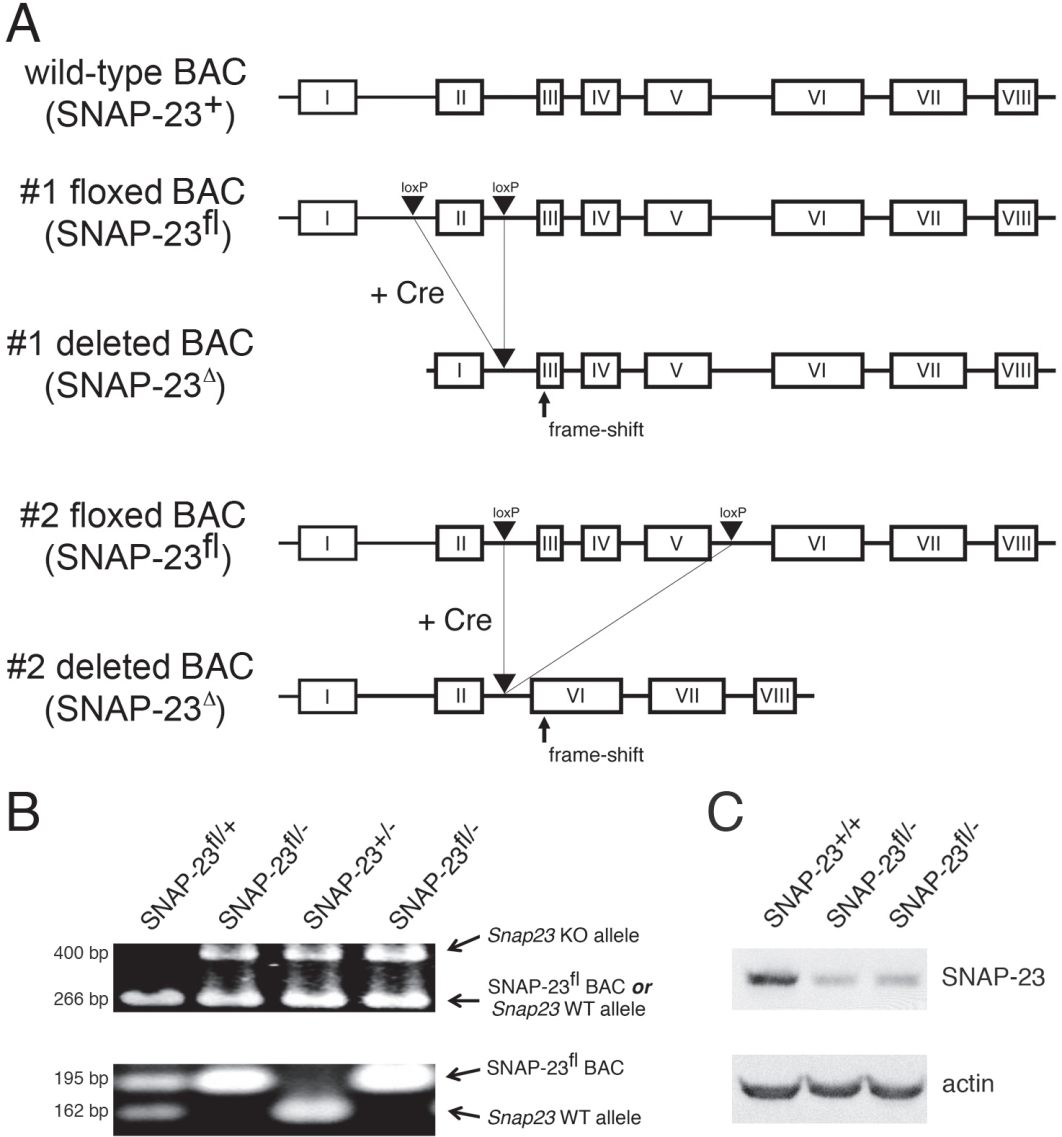
Generation of mice containing a floxed SNAP-23 allele. (A) A 179 kb BAC clone containing the entire SNAP-23 gene was mutated by introducing loxP sites surrounding exon II alone (#1 floxed allele) or surrounding exons III-V (#2 floxed allele). Expression of Cre will result in the excision of exon II alone in the #1 floxed allele construct or exons III-V in #2 floxed allele construct. Each deleted allele introduces a frame shift that prevents the synthesis of in-frame SNAP-23 exon-encoded amino acids. (B) Typical PCR genotyping analysis of mice used in this study. The upper panel shows results obtained using a PCR reaction that can distinguish the deleted SNAP-23 allele present in SNAP-23^+/-^ mice (492 bp) from either the wild-type SNAP-23 allele or the floxed SNAP-23 BAC transgene (266 bp). The lower panel shows results obtained using a PCR reaction that can distinguish the floxed SNAP-23 BAC transgene (195 bp) from the wild-type SNAP-23 allele present in SNAP-23^+/-^ mice (161 bp). (C) Spleen cells were isolated from wild-type mice (SNAP-23^+/+^) and two representative floxed SNAP-23 BAC transgene mice on a SNAP-23-/- background (SNAP-23^fl/-^) and the cells were lysed and analyzed by SDS-PAGE and immunobloting with SNAP-23 and actin antibodies. Quantitative densitometry revealed that the floxed SNAP-23 BAC expression of SNAP-23 was approximately 50% that found in wild-type mice.

Deletion of SNAP-23 leads to early (pre-implantation) embryonic lethality in mice [12]. Because of this we have been unable to determine whether the embryos die because SNAP-23 is required for generation/function/survival of specific embryonic cell (such as a trophoblast) or whether SNAP-23 is an essential protein that is required for the survival of all cells. To begin to address this question, we have generated BAC transgenic mice expressing forms of SNAP-23 in which either exon 2 alone or exons 3–5 were flanked by loxP sites (Fig. 1A). Mice harboring the floxed SNAP-23 BAC transgene were crossed onto a SNAP-23^+/-^ background, and these mice were then bred with SNAP-23^+/-^ mice to yield floxed SNAP-23 BAC^+^ mice on a SNAP-23-null background. All mice were genotyped using PCR primers that distinguish between the wild-type and deleted SNAP-23 alleles in SNAP-23^+/-^ mice and the floxed SNAP-23 BAC transgene (Fig. 1B). Immunoblot analysis of these mice revealed that the floxed SNAP-23 BAC (when present on a SNAP-23^-/-^ background) was expressed at approximately 50% the level found in wild-type mice (Fig. 1C). Despite the reduced expression of SNAP-23 in these mice, experiments comparing SNAP-23^fl/-^ mice to wild-type mice did not reveal any cellular or immunological defects in any of the assays performed in this study. Most importantly, expression of the SNAP-23 transgene completely rescues the early embryonic lethality observed in SNAP-23^-/-^ embryos and intercrossing floxed BAC^+^ SNAP-23^+/-^ mice gives us floxed BAC^+^ SNAP-23^+/+^, SNAP-23^+/-^, and SNAP-23^-/-^ mice with a normal Mendelian frequency. All mice used in this study were floxed SNAP-23 BAC^+^, and these mice are either on a SNAP-23^+/-^ background (termed SNAP-23^fl/+^ mice) or on a SNAP-23^-/-^ background (termed SNAP-23^fl/-^ mice).

### Deletion of SNAP-23 in CD19-Expressing Cells Prevents B Cell Development

**Fig 2:**
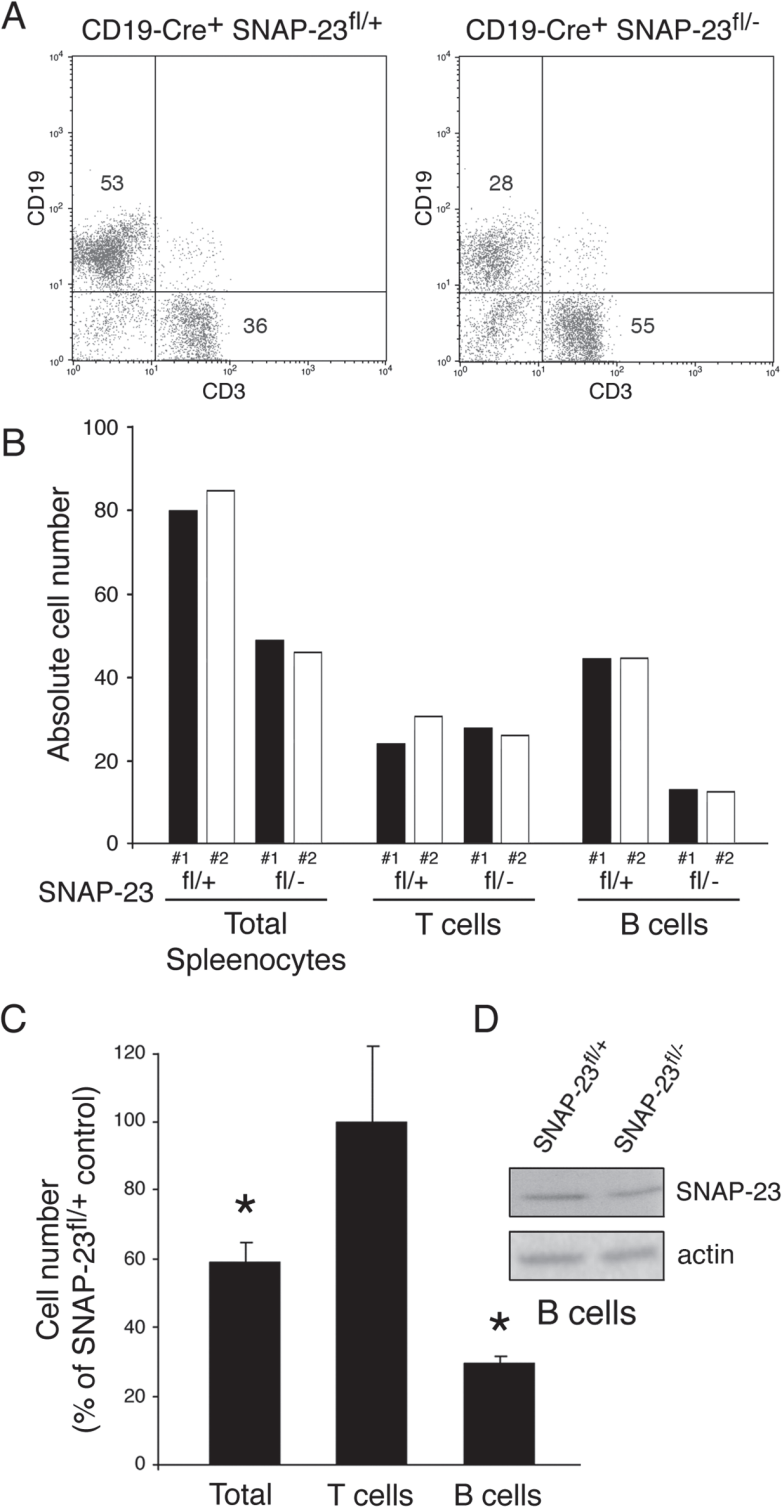
SNAP-23 is essential for B cell survival. Spleens were harvested from CD19-Cre^+^ SNAP-23^fl/+^ control mice or CD19-Cre^+^ SNAP-23^fl/-^ mice (line E3) and single cell suspensions were stained with fluorochrome-conjugated CD19- and CD3-mAb and analyzed by flow cytometry. (A) A representative flow cytometry profile reveals a dramatic reduction in the percentage of CD19^+^ B cells in the spleen. (B) Quantitative analysis of two different matched pairs of CD19-Cre^+^ SNAP-23^fl/+^ or SNAP-23^fl/-^ littermate mice indicating total spleen cell numbers as well as the numbers of T cells (CD3^+^ cells) and B cells (CD19^+^ cells) present in each spleen (calculated based on the percentage of each cell type present as determined by flow cytometry). (C) Summary of three independent experiments showing the recovery of the indicated cell types from the spleens of CD19-Cre^+^ SNAP-23^fl/-^ mice expressed as a percentage of those found in CD19-Cre^+^ SNAP-23^fl/+^ control mouse spleens. The data shown are mean +/- SD of three independent experiments (*p<0.05). (D) B cells were purified from the spleens from the indicated mice and equal numbers of cell equivalents were analyzed by SDS-PAGE and immunoblotting using a SNAP-23 antibody. The blot was re-probed for anti-β actin mAb as a loading control.

To investigate the importance of SNAP-23 in different cell types, we have used Cre-mediated excision to selectively remove either exon II or exons III-V from SNAP-23 by crossing these mice with SNAP-23^+/-^ mice expressing Cre recombinase under the control of different promoters. Cre-mediated excision in each construct results in a reading frame-shift that prevents expression of the SNAP-23 protein. It is important to note that we have never noted any differences in results obtained analyzing mice expressing either the #1 floxed BAC construct (targeting exon II) or the #2 floxed BAC construct (targeting exons III-V). Mice expressing Cre driven by the CD19 promoter express Cre throughout B cell development [13]. When we crossed CD19-Cre/SNAP-23^+/-^ mice with SNAP-23^fl/+^ mice we found that whereas CD19-Cre^+^/SNAP-23^fl/+^ pups had normal T cell and B cell profiles in the spleen, B cell numbers were dramatically reduced in CD19-Cre^+^/SNAP-23^fl/-^ pups (Fig. 2A, 2B). Quantitative analysis of spleen cell phenotype in multiple mice revealed that CD19-Cre^+^/SNAP-23^fl/-^ mice had less than 30% the number of B cells as compared to control CD19-Cre^+^/SNAP-23^fl/+^ mice (Fig. 2C). The viable B cells in these mice still possessed SNAP-23 protein, however, consistent with variegated expression of Cre-transgenes in mice. These data demonstrate that expression of Cre in spleen B cells (using the CD19 promoter) results in a specific, profound reduction of B cell numbers in SNAP-23^fl/-^ mice. Since we were unable to obtain live B cells lacking SNAP-23 in these mice, these data strongly suggest that expression of SNAP-23 is essential for B cell development and/or survival.

### Deletion of SNAP-23 in Lck-Expressing Cells Prevents T Cell Development

**Fig 3:**
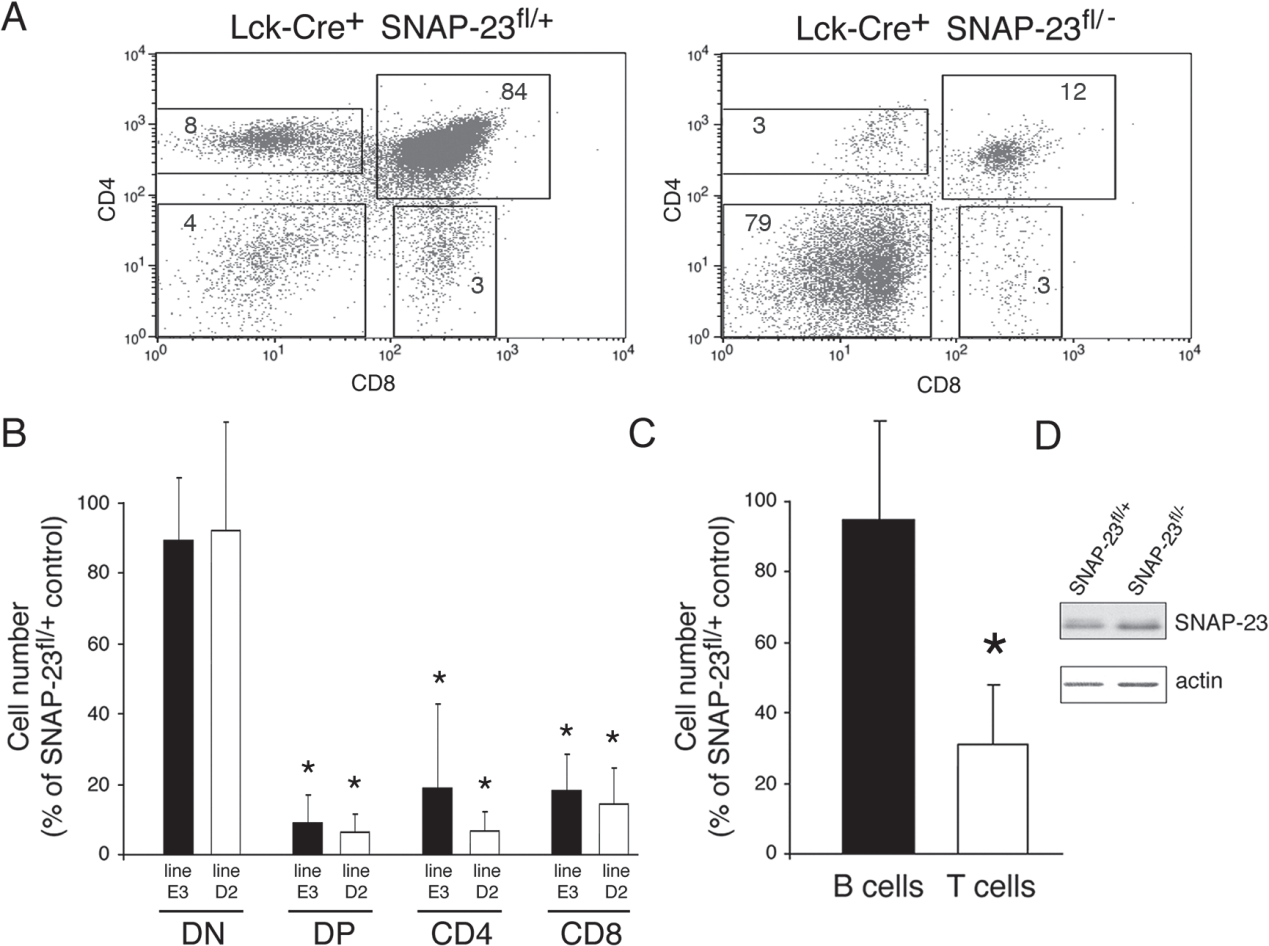
Deletion of SNAP-23 in Lck-expressing T cells prevents T cell development. Thymi were harvested from Lck-Cre^+^ SNAP-23^fl/+^ control mice or Lck-Cre^+^ SNAP-23^fl/-^ mice and single cell suspensions were stained with fluorochrome-conjugated CD4- and CD8-mAb and analyzed by flow cytometry. (A) A representative flow cytometry profile reveals a dramatic reduction in the percentage of CD4^+^, CD4^+^8^+^, and CD8^+^ T cells in the thymus of an Lck-Cre^+^-expressing SNAP-23^fl/-^ mouse (line D2). (B) Quantitative analysis of matched pairs of littermate mice from SNAP-23^fl^ founder lines E3 or D2 indicate recovery of CD4^-^CD8^-^ (DN), CD4^+^CD8^+^ (DP), CD4^+^, and CD8^+^ T cells present in thymi of Lck-Cre^+^ SNAP-23^fl/-^ mice expressed as a percentage of those found in thymi of Lck-Cre^+^ SNAP-23^fl/+^ control mice (calculated based on the percentage of each cell type present as determined by flow cytometry). The data shown are mean +/- SD of three independent experiments (*p<0.05). (C) The recovery of the indicated cell types from the spleens of Lck-Cre^+^ SNAP-23^fl/-^ mice was expressed as a percentage of those found in Lck-Cre^+^ SNAP-23^fl/+^ control mouse spleens (line D5). The data shown are mean +/- SD of eight independent experiments (*p<0.05). (D) T cells were purified from the spleens from the indicated mice and equal numbers of cell equivalents were analyzed by SDS-PAGE and immunoblotting using a SNAP-23 antibody. The blot was re-probed for anti-β actin mAb as a loading control.

The different stages of T cell development in the thymus can be identified by the expression of CD4 and CD8 proteins (reviewed in [14]). Early thymocytes do not express CD4 or CD8 proteins (termed DN cells). DN cells in the thymus proliferate and are signaled to express both CD4 and CD8 proteins on their surface (termed DP thymocytes). The process of positive selection ultimately results in the selective expression of either CD4 or CD8 protein, leading to the generation of CD4 or CD8 T cells, respectively [14]. T cell development requires intracellular signaling by the T cell kinase lck. Cre whose expression is controlled by the lck kinase proximal promoter is present very early in T cell development in DN thymocytes [15]. Lck-Cre is not expressed until the DN2 stage of T cell development and these cells rapidly proliferate and differentiate into DP cells. If deletion of SNAP-23 by Cre expressed by the lck proximal promoter leads to cell death, we predict that ablation of SNAP-23 in DN2 thymocytes will result in relatively normal amounts of DN cells but dramatically reduced amounts of DP, CD4, and CD8 T cells. In agreement with this prediction, we found that expression of lck-Cre in SNAP-23^fl/-^ mice resulted in a 10-fold reduction in the absolute numbers of DP, CD4, and CD8 T cells in two independent mouse lines (Fig. 3A, 3B). By contrast, the absolute number of DN thymocytes in lck-Cre^+^/SNAP-23^fl/-^ mice was only modestly reduced as compared to those present in lck-Cre^+^/SNAP-23^fl/+^ control mice.

Analysis of the spleens of these mice revealed normal numbers of B cells and a 70% reduction in T cells in lck-Cre^+^/SNAP-23^fl/-^ mice as compared to lck-Cre^+^/SNAP-23^fl/+^ control mice (Fig. 3C). As we observed in SNAP-23^fl/-^ mice expressing CD19-Cre, the small number of viable spleen T cells in lck-Cre^+^/SNAP-23^fl/-^ mice expressed normal amounts of SNAP-23 protein (Fig. 3D), a finding that is consistent with the idea that deletion of SNAP-23 in T cells results in a developmental block/death of T cells and that any surviving T cells in these mice escaped deletion of SNAP-23 by Cre.

### Deletion of SNAP-23 in CD8-Expressing DP Thymocytes Cells Prevent Development of CD4 and CD8 T Cells

**Fig 4:**
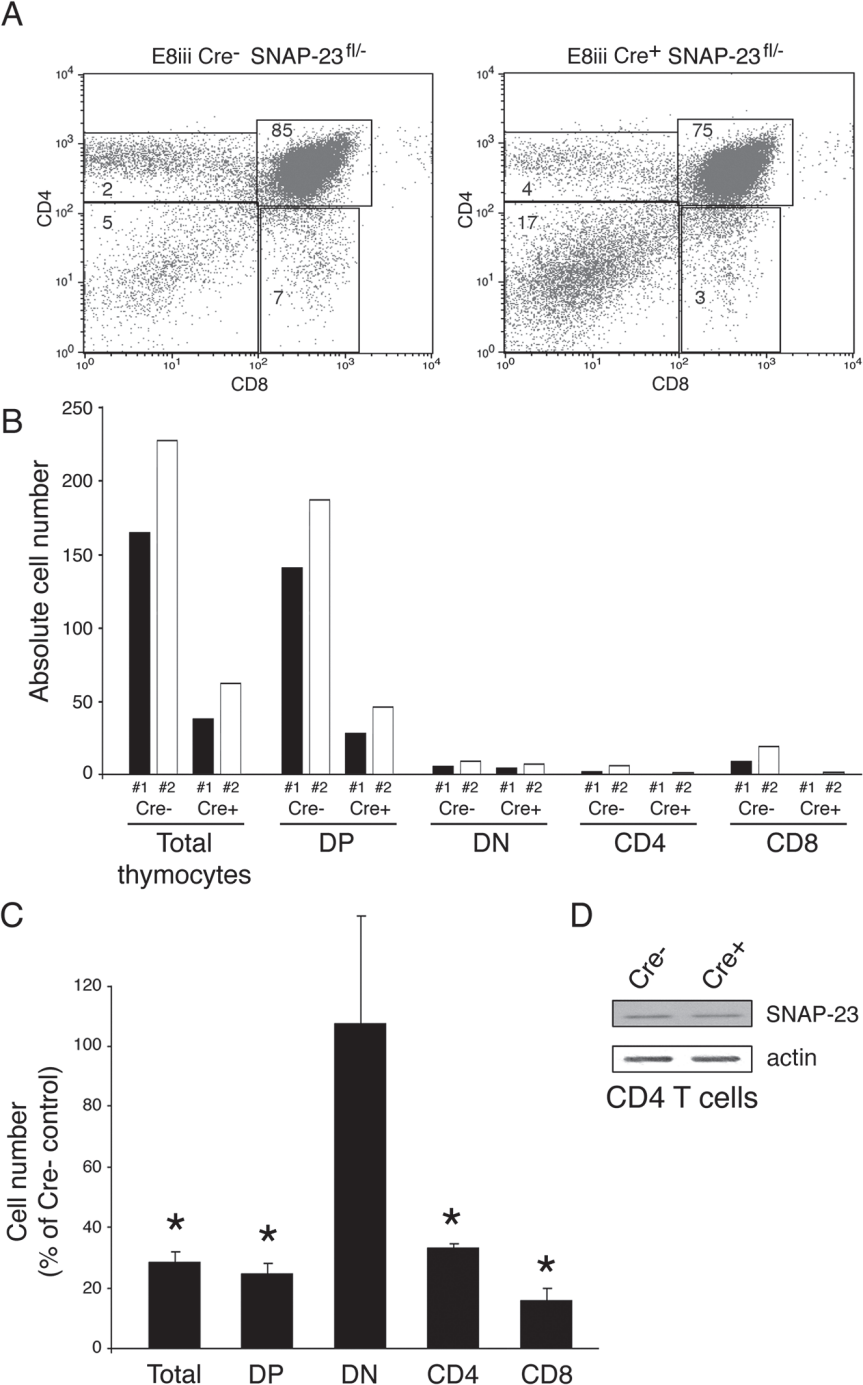
Deletion of SNAP-23 in CD4^+^CD8^+^ T cells prevents T cell development. Thymi were harvested from E8iii-Cre^-^ SNAP-23^fl/-^ control mice or E8iii-Cre^+^ SNAP-23^fl/-^ mice (line E3) and single cell suspensions were stained with fluorochrome-conjugated CD4- and CD8-mAb and analyzed by flow cytometry. (A) A representative flow cytometry profile reveals a dramatic reduction in the percentage of CD4^+^, CD4^+^8^+^, and CD8^+^ T cells in the thymus of an E8iii-Cre^+^-expressing SNAP-23^fl/-^ mouse. (B) Quantitative analysis of matched pairs of Cre^-^ and Cre+ SNAP-23^fl/-^ littermate mice lines indicate recovery of CD4^-^CD8^-^ (DN), CD4^+^CD8^+^ (DP), CD4^+^, and CD8^+^ T cells present in thymi of E8iii-Cre^+^ SNAP-23^fl/-^ mice expressed as a percentage of those found in thymi of E8iii-Cre^-^ SNAP-23^fl/-^ control mice (calculated based on the percentage of each cell type present as determined by flow cytometry). (C) The recovery of the indicated cell types from the thymi of E8iii-Cre^+^ SNAP-23^fl/-^ mice was expressed as a percentage of those found in E8iii-Cre^-^ SNAP-23^fl/-^ control mouse spleens. The data shown are mean +/- SD of five independent experiments (*p<0.05). (D) CD4 T cells were purified from the thymi from the indicated mice and equal numbers of cell equivalents were analyzed by SDS-PAGE and immunoblotting using a SNAP-23 antibody. The blot was re-probed for anti-β actin mAb as a loading control.

DP thymocytes and CD8 single positive T cells express CD8 under the control of two distinct promoters: DP thymocytes use the E8iii promoter to drive CD8 expression whereas mature CD8 T cells use the E8i promoter for CD8 expression [16]. We have therefore used E8iii-Cre mice and E8i-Cre mice [17] to examine the role of SNAP-23 deletion on DP thymocytes (and their progeny) or CD8 T cells, respectively. Expression of E8iii-Cre resulted in a profound block in T cell development and led to a 5-fold reduction in the numbers of total thymocytes, DP thymocytes, CD4 T cells, and CD8 T cells (Fig. 4A-C). As was observed in Lck-Cre mice, the numbers of DN thymocytes was unaffected by the E8iii-Cre transgene in SNAP-23^fl/-^ mice. Immunoblot analysis confirmed that the live CD4 T cells in E8iii-Cre^+^/SNAP-23^fl/-^ mice expressed SNAP-23 protein (Fig. 4D).

### Deletion of SNAP-23 in CD8 T Cells Prevents Development of CD8 but not CD4 T Cells

**Fig 5:**
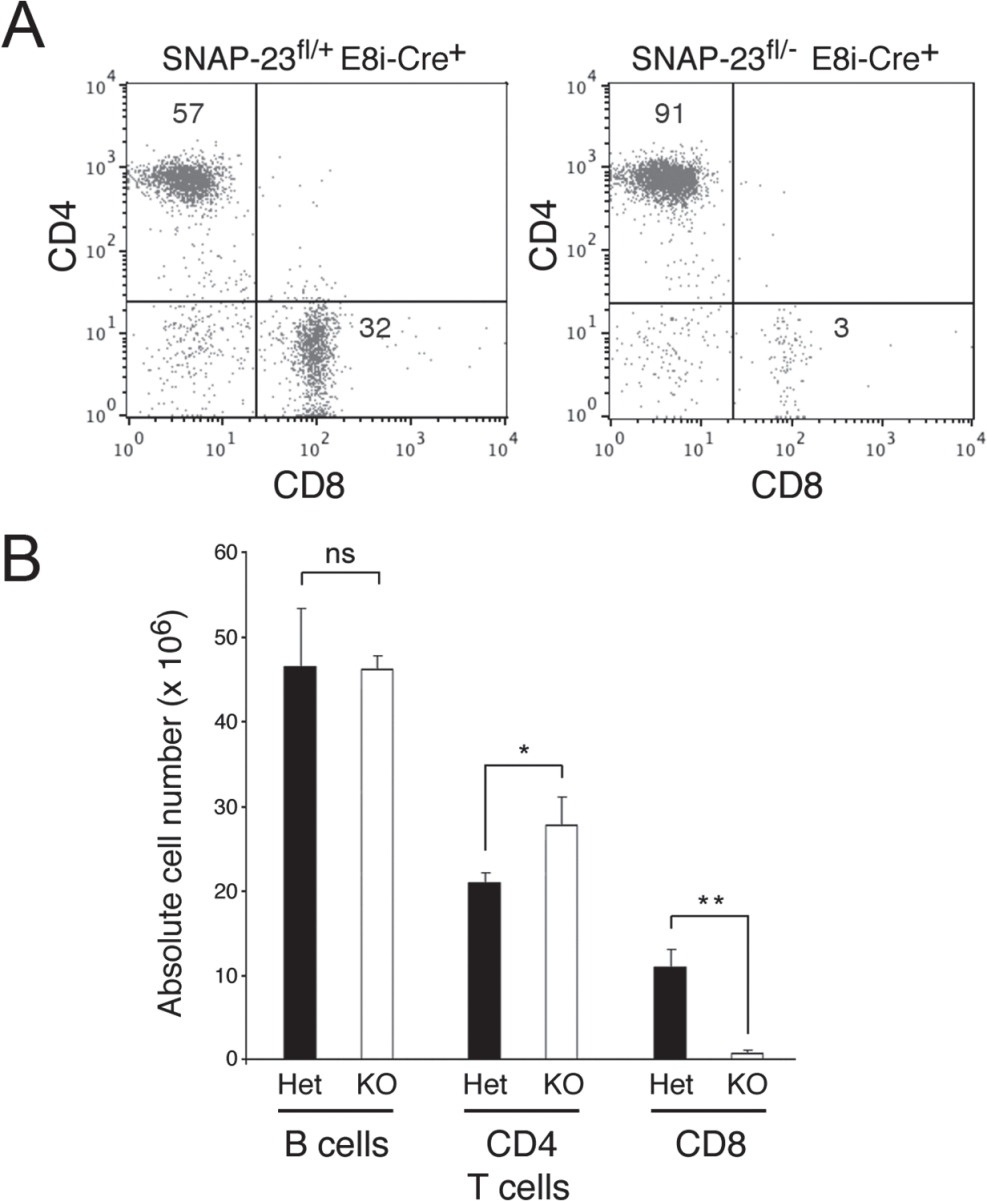
Deletion of SNAP-23 in thymus-derived CD8 T cells prevents survival of mature CD8 T cells in the spleen. Spleens were harvested from E8i-Cre^+^ SNAP-23^fl/+^ control mice or E8i-Cre^+^ SNAP-23^fl/-^ mice (line E3) and single cell suspensions were stained with fluorochrome-conjugated CD3-, CD4-, and CD8-mAb and analyzed by flow cytometry. (A) A representative flow cytometry profile of CD3-gated spleen cells reveals a selective reduction in the percentage of CD8^+^ T cells in the spleen. (B) Quantitative analysis of E8i-Cre^+^ SNAP-23^fl/+^ or E8i-Cre^+^ SNAP-23^fl/-^ littermate mice indicating total spleen cell numbers, B cell numbers (determined by gating on B220^+^ spleen cells) as well as the numbers of CD4 T cells and CD8 T cells present in each spleen (calculated based on the percentage of each cell type present as determined by flow cytometry). The data shown are mean +/- SD of three independent experiments (*p<0.05; **, p<0.01).

Expression of CD8 in mature, single positive CD8 T cells is regulated by the E8i promoter [16]. When Cre expression was regulated by the E8i promoter, we observed almost complete absence of CD8 T cells in the spleen (Fig. 5A, B). In these E8i-Cre^+^/SNAP-23^fl/-^ spleens we found normal numbers of B cells and slightly elevated numbers of CD4 T cells. Taken with our results obtained above, these data show that expression of Cre by a promoter that leads to preferential expression in mature CD8 T cells (E8i) selectively deletes these cells whereas expression of Cre by a more ubiquitious CD8 promoter (E8iii) leads to a profound block in development/survival of both CD4 and CD8 T cells.

### Acute Depletion of SNAP-23 in Fibroblasts Leads to Apoptotic Cell Death

**Fig 6:**
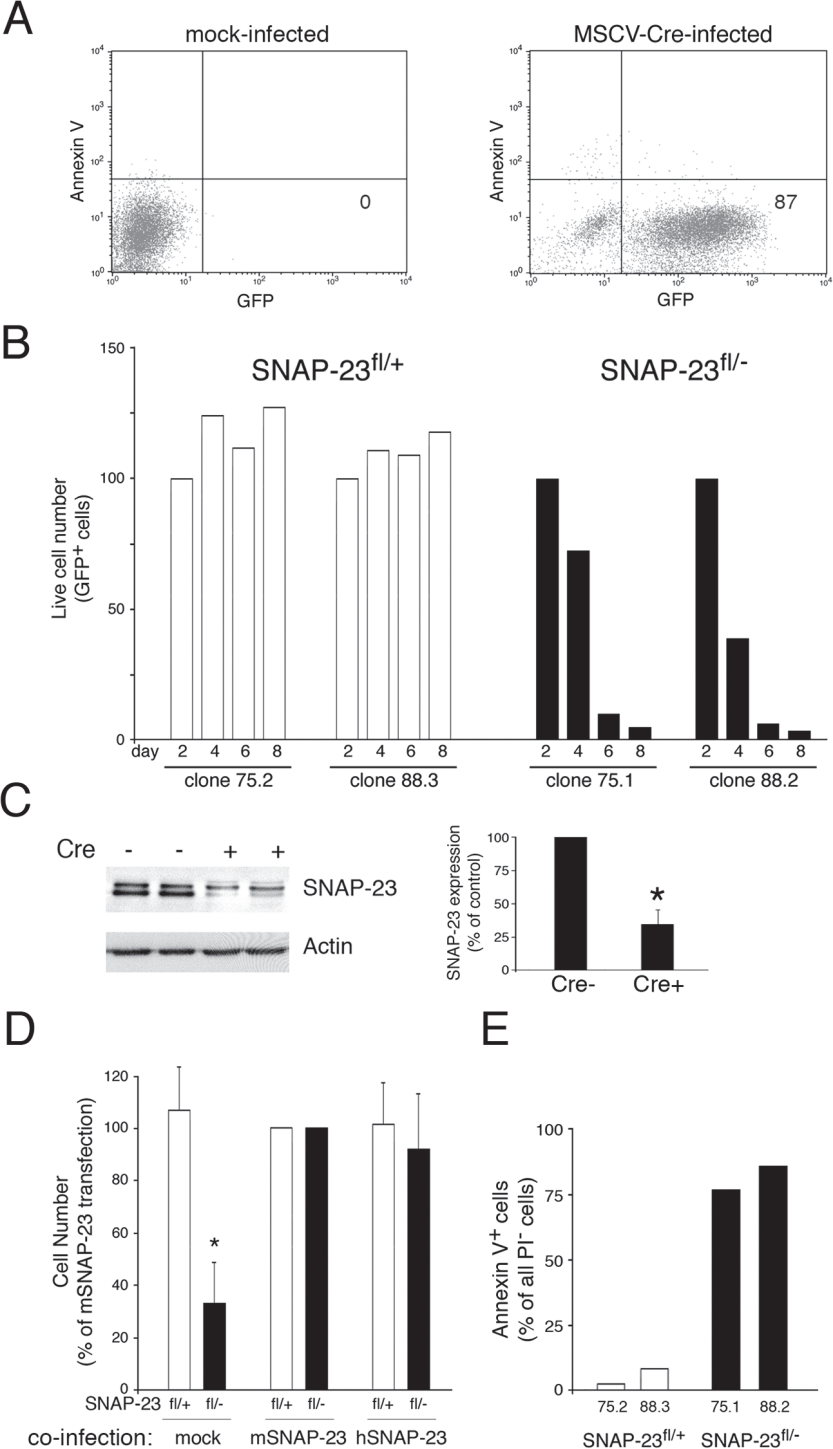
Deletion of SNAP-23 leads to acute death of MEFs. MEF lines were generated from SNAP-23^fl/+^ mice (clones 75.1 and 88.2) or SNAP-23^fl/-^ mice (clones 75.2 and 88.3). (A) Infection of a typical SNAP-23^fl/+^ MEF culture infected with empty retrovirus (left panel) or GFP-Cre retrovirus (right panel) shows that two days after infection the vast majority of MEFs were viable (Annexin V^-^) and expressed GFP-Cre. (B) The indicated MEF lines were infected with GFP-Cre-expressing retrovirus and the number of live cells present in each culture (based on staining with PI) was determined at different times. The absolute cell recovery in each condition was expressed relative to the amount of cells present two days after infection (control experiments showed that there was no Cre-dependent cell death in any line after only two days of infection). The data shown are representative of two independent experiments analyzed at day 2, 4, 6, 8 and one experiment analyzed at day 1, 3, 5, 7. (C) Adherent SNAP-23^fl/-^ MEFs were isolated 4 days after retroviral transduction with Cre (Cre+) or after mock-transduction (Cre-). Equal numbers of cells from each culture were analyzed by SDS-PAGE and immunoblotting using a SNAP-23 antibody. The blot was re-probed for anti-β actin mAb as a loading control. The amount of SNAP-23 present in each cell lysate was normalized to the amount of actin present and the data shown are mean +/- SD of three independent experiments (*p<0.05). (D) MEF lines generated from SNAP-23^fl/+^ mice or SNAP-23^fl/-^ mice were co-infected with retrovirus containing GFP-Cre and retrovirus containing nothing (mock), mouse SNAP-23 (mSNAP-23), or human SNAP-23 (hSNAP-23). After 4 days the number of live cells present in each culture (based on staining with PI) was determined and was expressed relative to the number of cells recovered in the mSNAP-23 co-infection condition. The data shown are mean +/- SD of four independent experiments (*p<0.05). (E) The indicated MEF lines were infected with GFP-Cre-expressing retrovirus and after four days the cells were stained with PI and Annexin V and analyzed for GFP-Cre expression. The percentage of viable (PI^-^) GFP-Cre^+^ Annexin V^+^ cells in each culture was determined by flow cytometry. The data shown are representative of three independent experiments.

We next tested the hypothesis that SNAP-23 expression is essential for cell survival by acutely depleting SNAP-23 in mouse embryonic fibroblasts (MEFs) isolated from SNAP-23^fl/-^ mice. Multiple MEF clones were isolated from SNAP-23^fl/-^ mice or SNAP-23^fl/+^ control mice and these cells were infected with control (empty) retrovirus or retrovirus expressing GFP and Cre. Four days after transduction with the control retrovirus we found that greater than 90% of all SNAP-23^fl/+^ MEFs expressed GFP (Fig. 6A), indicating the high efficiency of retroviral transduction in these cells. When the survival of MEF clones isolated from SNAP-23^fl/+^ and SNAP-23^fl/-^ mice were compared we found that acute expression of Cre resulted in profound death of essentially all GFP^+^ SNAP-23^fl/-^ MEFs during one week of culture (as indicated by a reduction in propidium iodide (PI)^-^ viable cells; Fig. 6B). Note that retroviral transduction inhibited the growth of even SNAP-23^fl/+^ MEFs but unlike MEFs isolated from SNAP-23^fl/-^ mice, the MEFs from SNAP-23^fl/+^ mice did not die during the one-week culture period. To confirm that the death of Cre-expressing SNAP-23^fl/-^ MEFs was due to acute reduction of SNAP-23 protein levels, we analyzed SNAP-23 expression in the adherent cells remaining 4 days after transduction of SNAP-23^fl/-^ MEFs with Cre. When we compared the amount of SNAP-23 present in these cells to the amount present in mock-transduced cells we found a 65% reduction in SNAP-23 protein (Fig. 6C). As anticipated, the death of the SNAP-23^fl/-^ MEF cell lines was prevented by overexpressing retroviral constructs encoding either mouse SNAP-23, human SNAP-23, or mouse SNAP-25 prior to Cre-mediated deletion of SNAP-23 (Fig. 6D). The death of SNAP-23^fl/-^ MEFs absolutely required expression of Cre, as the small numbers of GFP^-^ (i.e. untransduced) MEFs in the SNAP-23^fl/-^ MEF culture did not die during the week-long culture period (S1 Fig.).

Although we classified PI^-^ cells as “alive”, these cells were not necessarily healthy and could be in the process of cell death. To determine if the PI^-^ cells observed in this study were undergoing apoptotic cell death, we counterstained the cells with the apoptotic cell marker Annexin V. When the small numbers of viable GFP^+^ PI^-^ cells present 8 days after retroviral transduction of SNAP-23^fl/-^ MEFs were analyzed with Annexin-V we found that nearly all of the remaining “live” cells were Annexin-V^+^ and were thus in the process of apoptotic cell death (Fig. 6E). By contrast, almost none of the GFP^+^ PI^-^ cells in the culture of transduced SNAP-23^fl/+^ control MEFs were undergoing apoptosis. Taken together, these studies show that SNAP-23 is an essential gene and that acute depletion of SNAP-23 results in apoptotic cell death.

Apoptotic cell death leads to nuclear fragmentation, a process that releases genomic DNA and often leads to cells “clumping” together during apoptosis. We therefore performed time-lapse video microscopy to visualize the effects of GFP-Cre on MEF cell viability/morphology. During the 30 hour time course of imaging on a 37°C stage we readily observed SNAP-23^fl/-^ MEFs transduced with GFP-Cre retrovirus “shriveling up” and GFP^+^ cells clumping together (S1 Video, S2 Video). By contrast, non-transduced (GFP^-^) SNAP-23^fl/-^ MEFs in the same field appear healthy as did mock-infected SNAP-23^fl/-^ MEFs (S3 Video, S4 Video). Similarly, SNAP-23^fl/+^ control MEFs expressing GFP-Cre appeared healthy through the time course of study (S5 Video, S6 Video). While these studies do not unambiguously reveal the mechanism of MEF cell death, the results observed demonstrate that the loss of cells observed in this study are due to cell death and are consistent with the hypothesis that depletion of SNAP-23 leads to apoptotic cell death.

Germline deletion of SNAP-23 leads to embryonic lethality very early in mouse embryo development, with SNAP-23-null embryos dying prior to implantation in the uterus [12]. The reason for this could be that SNAP-23 is important for a protein transport process that is specifically required for an embryonic cell development pathway that is essential for blastocyst viability (such as GLUT8 transporter, [18]), or alternatively it is possible that SNAP-23 expression is absolutely required for viability of any cell type. Given the wide variety of protein trafficking steps that have been shown to be regulated by SNAP-23 [4–11], we felt it was likely that deletion of SNAP-23 would lead to acute cell death. By creating mice with a floxed SNAP-23 allele we have been able to generate “normal” mice in which SNAP-23 is only deleted in cells expressing Cre recombinase under the control of various cell-specific promoters. Our results show that deletion of SNAP-23 in B cells (by using CD19-Cre) or in T cells (by using three different T cell-specific Cre mice) leads to the selective elimination of the Cre expressing cell subtype. In each case, the small numbers of cells remaining in such mice still express SNAP-23, a finding that is consistent with variegated Cre transgene expression in mice. These results strongly suggested that SNAP-23 expression is essential for cell viability. We tested this hypothesis by acutely deleting SNAP-23 in MEF cells expressing SNAP-23^fl/-^ alleles, and this treatment resulted in the rapid death of the MEFs by apoptosis. Taken together, these data show that SNAP-23 is an essential gene. Given the known role of SNAP-23 in the process of regulated exocytosis and vesicle fusion, it is likely that SNAP-23 deletion leads to cell death by preventing an essential vesicle trafficking/cargo secretion event. Unfortunately, our inability to isolate viable cells that do not express SNAP-23 makes testing this hypothesis impossible.

## Materials and Methods

### Mice

BAC RP23-377I14 is a 179-kilobase (kb) BAC containing 74 kb of sequence upstream and 72.2 kb sequence downstream of the SNAP-23 gene, respectively. Lox-P sites flanking either exon 2 (construct#1) or exon 3–5 (construct#2) were introduced into this BAC using oligonucleotide-based recombination [19]. The sequences of the oligonucleotides used to generate these constructs as well as the primers used to identify the presence of the BAC transgene in mice are described in S1 Materials and Methods.

SNAP-23^+/-^ mice have been described previously and all pups were genotyped using previously described primers [12]. CD19-Cre transgenic mice [13], Lck proximal promoter-Cre transgenic mice [15], and E8iii-Cre transgenic mice [17] have been described. CD19-Cre transgenic mice were obtained from Richard Hodes (National Cancer Institute, National Institutes of Health). E8i-Cre mice were generated by Amala Alag, Batu Erman, and Alfred Singer (National Cancer Institute, National Institutes of Health) and generously provided prior to publication. Lck-Cre and E8iii-Cre transgenic mice were also obtained from Alfred Singer (National Cancer Institute, National Institutes of Health). The presence of each Cre recombinase transgene in pups was determined by PCR using primers described in S1 Materials and Methods. Floxed SNAP-23 BAC mice were bred with SNAP-23^+/-^ mice to generate floxed BAC^+^ SNAP-23^+/-^ mice (termed SNAP-23^fl/+^ mice). SNAP-23^+/-^ mice were also bred with different Cre-transgenic mice to obtain Cre^+^ SNAP-23^+/-^ mice. These mice were bred with SNAP-23^fl/+^ mice to generate to generate cell-specific knockout of the SNAP-23 gene contained within the BAC. This study was carried out in strict accordance with the recommendations in the Guide for the Care and Use of Laboratory Animals of the National Institutes of Health. The protocol was approved by the National Cancer Institute Animal Care and Use Committee (Protocol #EIB-076).

### Virus Production and MEF Infection

A mouse stem cell virus (MSCV) vectors expressing Cre recombinase together with GFP [20] was provided by Anne Gegonne (NCI, NIH). MSCV expressing mouse SNAP-23 or human SNAP-23 together with the nerve growth factor receptor (NGFR) were generated by inserting the appropriate PCR amplified form of SNAP-23 into the Bgl II and EcoR I restriction sites of the MSCV-IRES-NGFR vector [21] (kindly provided by Anne Gegonne, NCI, NIH). MSCV was prepared in ecotropic PlatE cells (Cell Biolabs) using Lipofectamine 2000 (Invitrogen) as described [22].

SNAP-23^fl/+^, or SNAP-23^fl/-^ MEFs were infected with either MSCV-GFP-Cre alone or co-infected with MSCV-GFP-Cre and either MSCV-NGFR (control) or MSCV-SNAP-23-NGFR in presence of Polybrene and cultured at 37°C in DMEM medium containing 10% FBS as described [22]. At various times after infection, cells were harvested, counted, incubated with 10 μg/ml Propidium Iodide (PI) and analyzed by FACS. Based on the percentage of GFP^+^ PI^-^ cells observed in FACS analysis and the absolute number of cells in the culture we determined the absolute number of viable infected cells present in each culture.

### Purification of B Cells and CD4+ T cells from Spleen and Western Blotting

Spleens were harvested from mice and CD4^+^ T cells or total B cells were purified from single-cell suspensions by magnetic cell sorting using the appropriate MACS Cell Isolation Kit according to the manufacturers protocols (Miltenyi Biotec). The cell purity was routinely >90% pure following specific cell isolation (as determined by FACS analysis). Cells were lysed by incubation in 1X SDS-PAGE sample buffer for 3 min at 95°C followed by brief sonication to shear DNA. SDS-PAGE and immunoblotting was performed as described [23]. The amount of SNAP-23 protein present in cell lysates was determined by immunoblotting using a rabbit anti-mouse SNAP-23 antibody [3] and quantitated on a Molecular Dynamics Densitometer and ImageQuant software (Sunnyvale, CA). The loading of the different samples was normalized by re-probing every blot with mouse monoclonal anti-β actin antibody (Millipore).

### Flow Cytometry

Single cell suspensions were obtained from freshly-harvested thymus or spleen and red blood cells were removed from the splenocytes preparation by ACK lysis. The cells were then stained on ice in FACS buffer (HBSS containing 2% FBS) using fluorochrome-conjugated antibodies. The anti-mouse CD3ε, TCR-β, CD4, CD8, B220 and CD19 mAb used in this study were purchased from BD Biosciences. The cells were also stained using the appropriate isotype control antibodies. The stained cells were acquired and analyzed by using FACS Calibur (BD Biosciences). The data was analyzed with FSC/SSC gating to exclude dead cells and cell debris from analysis. In all experiments analyzing MEFs, PI-staining was also used to differentiate dead from viable cells. In some experiments, cells were stained with PE-conjugated Annexin-V on ice to reveal apoptotic cells.

### Live Cell Microscopy

SNAP-23^fl/+^ or SNAP-23^fl/-^ MEFs were plated in a 2-well Nunc Lab-Tek Chambered Coverglass in a volume of 1 ml (50,000 cells/ml). The following day MEFs were infected with MSCV-GFP-Cre virus in presence of Polybrene. The cultures were incubated at 37°C in a 5% CO_2_ atmosphere and media was changed every other day. The cultures were moved to the microscope 4 days post-infection. Confocal time series images were collected using a Zeiss LSM510 Meta laser scanning confocal microscope equipped with a 20x Plan-apochromat (N.A. 0.8) objective lens and an environmental stage top incubator with temperature, humidity, and CO_2_ control. The 488 nm laser line from a multi-line Argon laser was used for imaging GFP^+^ cells with 0.25% of the output power used for excitation intensity. GFP^-^ cells were imaged using differential interference contrast microscopy. Images were acquired every 20 or 30 min over a time period of 30 hr. Each image in the time series was acquired with 0.44 μm X-Y pixel size and a 1.0 μm optical slice thickness, and exported as a video file using Zeiss Zen software and saved as a movie file (.mov) format at 5 frames per second.

## Supporting Information

S1 Fig SNAP-23^fl/-^ MEF lines do not die in the absence of GFP-Cre expression. MEF lines were generated from SNAP-23^fl/+^ mice (clones 75.1 and 88.2) or SNAP-23^fl/-^ mice (clones 75.2 and 88.3). The indicated MEF lines were infected with GFP-Cre-expressing retrovirus and the number of GFP^-^ (GFP-negative) live cells present in each culture (based on staining with PI) was determined at different times. The absolute cell recovery in each condition was expressed relative to the amount of cells present two days after. The data shown are average of two independent experiments analyzed at day 2, 4, 6, 8.
(TIF)

S1 Materials and Methods (DOC)

S1 Video SNAP-23fl/- MEF line 75.1 infected with GFP-Cre retrovirus. The SNAP-23fl/- MEF line 75.1 was infected with GFP-Cre retrovirus one day after plating the cells in glass bottom chambers. The infected MEF cultures were moved 4 days post-infection to the microscope equipped with an environmental stage top incubator with temperature, humidity and CO_2_ control. Images were acquired every 20 min over a period of 30 hr. The time series of images from each location was saved as a movie file.
(MOV)

S2 Video SNAP-23fl/- MEF line 75.1 infected with GFP-Cre retrovirus. The SNAP-23fl/- MEF line 75.1 was infected with GFP-Cre retrovirus one day after plating the cells in glass bottom chambers. The infected MEF cultures were moved 4 days post-infection to the microscope equipped with an environmental stage top incubator with temperature, humidity and CO_2_ control. Images were acquired every 20 min over a period of 30 hr. The time series of images from each location was saved as a movie file.
(MOV)

S3 Video SNAP-23fl/- MEF line 75.1 mock-infected. The SNAP-23fl/- MEF line 75.1 was mock-infected one day after plating the cells in glass bottom chambers. The infected MEF cultures were moved 4 days post-infection to the microscope equipped with an environmental stage top incubator with temperature, humidity and CO_2_ control. Images were acquired every 20 min over a period of 30 hr. The time series of images from each location was saved as a movie file.
(MOV)

S4 Video SNAP-23fl/- MEF line 75.1 mock-infected. The SNAP-23fl/- MEF line 75.1 was mock-infected one day after plating the cells in glass bottom chambers. The infected MEF cultures were moved 4 days post-infection to the microscope equipped with an environmental stage top incubator with temperature, humidity and CO_2_ control. Images were acquired every 20 min over a period of 30 hr. The time series of images from each location was saved as a movie file.
(MOV)

S5 Video SNAP-23fl/+ control MEF line 75.2 infected with GFP-Cre retrovirus. The SNAP-23fl/+ MEF line 75.2 was infected with GFP-Cre retrovirus one day after plating the cells in glass bottom chambers. The infected MEF cultures were moved 4 days post-infection to the microscope equipped with an environmental stage top incubator with temperature, humidity and CO_2_ control. Images were acquired every 30 min over a period of 30 hr. The time series of images from each location was saved as a movie file.
(MOV)

S6 Video SNAP-23fl/+ control MEF line 75.2 infected with GFP-Cre retrovirus. The SNAP-23fl/+ MEF line 75.2 was infected with GFP-Cre retrovirus one day after plating the cells in glass bottom chambers. The infected MEF cultures were moved 4 days post-infection to the microscope equipped with an environmental stage top incubator with temperature, humidity and CO_2_ control. Images were acquired every 30 min over a period of 30 hr. The time series of images from each location was saved as a movie file.
(MOV)
