# Supplementary figures and images for: Expression of the SNARE Protein SNAP-23 Is Essential for Cell Survival
Source: PLoS One. 2015 Feb 23;10(2):e0118311. doi: 10.1371/journal.pone.0118311 (PMC4338070; doi:10.1371/journal.pone.0118311)

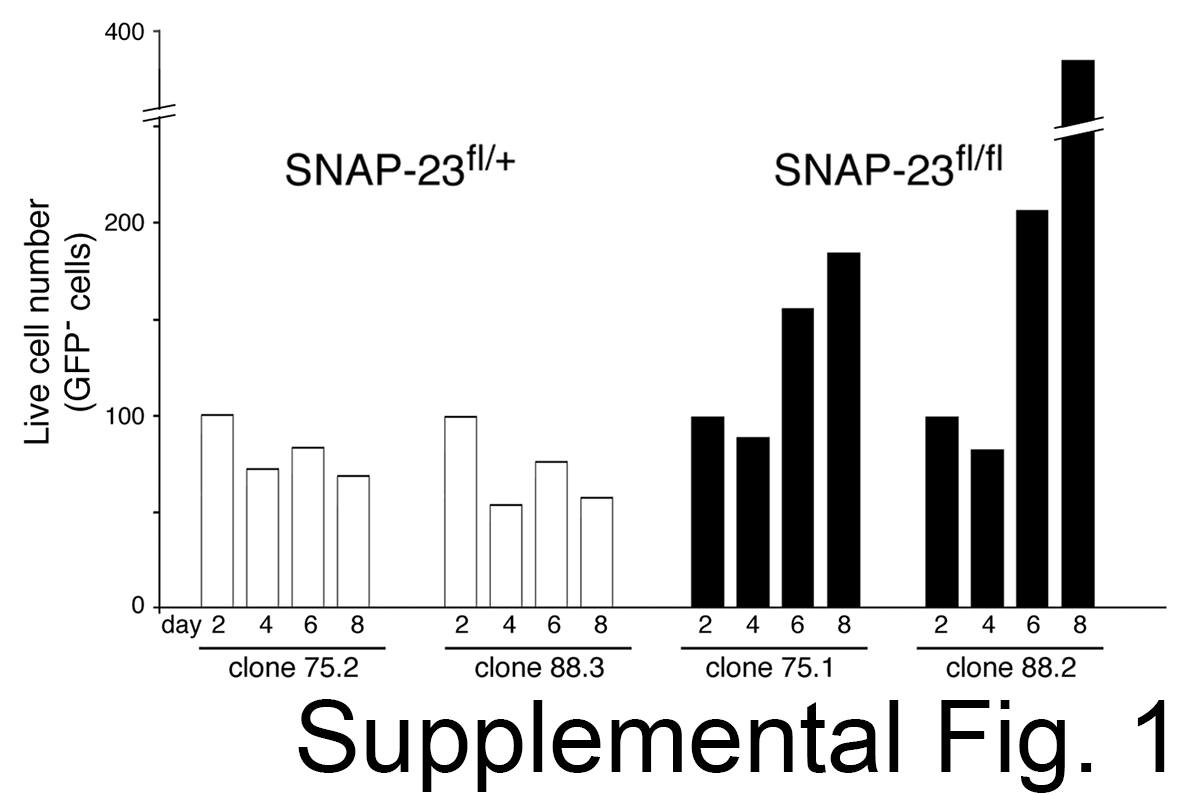

Supplement: S1 Fig — MEF lines were generated from SNAP-23fl/+ mice (clones 75.1 and 88.2) or SNAP-23fl/- mice (clones 75.2 and 88.3). The indicated MEF lines were infected with GFP-Cre-expressing retrovirus and the number of GFP- (GFP-negative) live cells present in each culture (based on staining with PI) was determined at different times. The absolute cell recovery in each condition was expressed relative to the amount of cells present two days after. The data shown are average of two independent experiments analyzed at day 2, 4, 6, 8. (TIF) [file pone.0118311.s001.tif]
