# Supplementary material for: Expression of the SNARE Protein SNAP-23 Is Essential for Cell Survival
Source: PLoS One. 2015 Feb 23;10(2):e0118311. doi: 10.1371/journal.pone.0118311 (PMC4338070; doi:10.1371/journal.pone.0118311)
Supplement: S1 Materials and Methods — (DOC) [file pone.0118311.s002.doc]

**Supporting Materials and Methods S1**

**Generation of SNAP-23^fl^ Mice**

BAC RP23-377I14 is a 179-kilobase (kb) BAC containing 74 kb of sequence upstream and 72.2 kb sequence downstream of the *Snap23* gene, respectively. Lox-P sites flanking either exon 2 (construct#1) or exon 3-5 (construct#2) were introduced into this BAC using oligonucleotide-based recombination. A Lox-P site was introduced in intron 2 (Target Site-2) and this construct containing this Lox-P site was used as the template to introduce an additional Lox-P site into intron 1 (Target Site-1) for construct#1 or into intron 5 (Target Site-3) for construct#2 (the sequences of the Lox-P sites are indicated by underline).

Target Site-1 Forward Primer: GCATGTGGAGGGCAGAGGACAAGTTTAAAAAAGTTTATTGGTGTTTGGGGGATGGCATGGAGATCAGAAGATAGCGTTGATAACTTCGTATAGCATACATTATACGAAGTTATTAGACTCCTG

Target Site-1 Reverse Primer: TTAAAATGCTTGCTATGCAAGTCTGACAACCTATGAGCACTGAAATGGCTTAACAGGTAAAGGAGCTTGCCAGGAGTCTAATAACTTCGTATAATGTATGCTATACGAAGTTATCAACGCTATC

Target Site-2 Forward Primer: GAGTACACCACCATTGCTCTCTTCAGACACACCAGAAGAGAGCACCAGATCCCATTATAGATGGTTATGAGCCACCATGTATAACTTCGTATAGCATACATTATACGAAGTTATGGTTGCTGGG,

Target Site-2 Reverse Primer: TCTCTGGGTTGGAAAGATGGCTCAGTGGTTAAGAGCACTGACTGATCTTCTAGAGGTCCTGAGTTCAATTCCCAGCAACCATAACTTCGTATAATGTATGCTATACGAAGTTATACATGGTGGC

Target Site-3 Forward Primer: AGGTTCATAGAAAATCGTGCCTAGATTGACCAACTCTTGTACCAAACATCAGTTTCAATCTGGATACAAAAAGACTGGGGATAACTTCGTATAGCATACATTATACGAAGTTATAATGTGCGTTTG,

Target Site-3 Reverse Primer: CCTATGAAGAAGACCATTCTTTACTCCTTGACTGTCTAGATACAAATGACATTCTGTTCTACATCATCCAAACGCACATTATAACTTCGTATAATGTATGCTATACGAAGTTATCCCCAGTC

**Mouse Genotyping**

The presence of the floxed *Snap23* BAC transgene was determined by screening tail DNA using the following PCR primer pairs for Target Site-1, PCR1-For: GCGTTGATAACTTCGTATAGCATAC, PCR1-Rev: CAGAGACAGGAACTCAGGCT (PCR product 474 bp), Target Site-2, PCR2- For: CACCATGTATAACTTCGTATAGC, PCR2- Rev: CCTCAATGGCTAAACCCAGG (PCR product 515 bp), or Target Site-3, PCR3-For: GACTGGGGATAACTTCGTATAGC, PCR3- Rev: CTAGCCTCCTGGTATAGTCTCGA (PCR product 465 bp).

The presence of wild-type or deleted endogenous *Snap23* alleles was assayed using PCR primer sets as described by Suh et al (2011). The presence of the wild-type *Snap23* allele in mice carrying the #1 floxed BAC transgene (D2 founder line) was determined by using the following primer set (GAGTACACCACCATTGCTCTC, CTCTGGGTTGGAAAGATGGC) and in mice carrying the #2 floxed BAC transgene (E3 and D5 founder lines) was determined by using the following primer set (ATAGCGTTGTAGACTCCT, CCAGCAACCACATGGTGG).

The presence of Cre recombinase transgene in the various mice used in this study was assayed by using the following PCR primer pairs: CD8 enhancer 1 (E8i) and CD8 enhancer 3 (E8iii) (cctggaaaatgcttctgtccgtttg, acgaacctggtcgaaatcagtgcg); Lck proximal promoter (CGATGCAACGAGTGATGAGG, GCATTGCTGTCACTTGGTCGT); and CD19 promoter (TGAGAAGCTGGCTTGGTATCGA, GTGAAACAGCATTGCTGTCACTT).
